# Host Immune Response to ZIKV in an Immunocompetent Embryonic Mouse Model of Intravaginal Infection

**DOI:** 10.3390/v11060558

**Published:** 2019-06-17

**Authors:** Svetlana F. Khaiboullina, Priscila Lopes, Toniana G. de Carvalho, Ana Luiza C. V. Real, Danielle G. Souza, Vivian V. Costa, Mauro M. Teixeira, Enrrico Bloise, Subhash C. Verma, Fabiola M. Ribeiro

**Affiliations:** 1 Department of Microbiology and Immunology, University of Nevada, Reno, NV 89557, USA; sv.khaiboullina@gmail.com; 2 Department of Biochemistry and Immunology, Institute of Biological Sciences (ICB), Universidade Federal de Minas Gerais, Belo Horizonte 31270-901, MG, Brazil; pripsgraz@gmail.com (P.L.); toniana_gc@hotmail.com (T.G.d.C.); analuza321@gmail.com (A.L.C.V.R.); mmtex.ufmg@gmail.com (M.M.T.); 3 Department of Microbiology, Institute of Biological Sciences (ICB), Universidade Federal de Minas Gerais, Belo Horizonte 31270-901, MG, Brazil; souzadg@gmail.com; 4 Department of Morphology, Institute of Biological Sciences (ICB), Universidade Federal de Minas Gerais, Belo Horizonte 31270-901, MG, Brazil; vivianvcosta@gmail.com (V.V.C.); enrico_bloise@hotmail.com (E.B.)

**Keywords:** ZIKV, FVB/NJ mice, intravaginal infection, CCL2, CXCL1 and CXCL10

## Abstract

Zika virus (ZIKV) only induces mild symptoms in adults; however, it can cause congenital Zika syndrome (CZS), including microcephaly. Most of the knowledge on ZIKV pathogenesis was gained using immunocompromised mouse models, which do not fully recapitulate human pathology. Moreover, the study of the host immune response to ZIKV becomes challenging in these animals. Thus, the main goal of this study was to develop an immunocompetent mouse model to study the ZIKV spread and teratogeny. FVB/NJ immune competent dams were infected intravaginally with ZIKV during the early stage of pregnancy. We found that the placentae of most fetuses were positive for ZIKV, while the virus was detected in the brain of only about 42% of the embryos. To investigate the host immune response, we measured the expression of several inflammatory factors. Embryos from ZIKV-infected dams had an increased level of inflammatory factors, as compared to Mock. Next, we compared the gene expression levels in embryos from ZIKV-infected dams that were either negative or positive for ZIKV in the brain. The mRNA levels of viral response genes and cytokines were increased in both ZIKV-positive and negative brains. Interestingly, the levels of chemokines associated with microcephaly in humans, including CCL2 and CXCL10, specifically increased in embryos harboring ZIKV in the embryo brains.

## 1. Introduction

Zika virus (ZIKV) is an arbovirus from the *Flaviviridae* family that is transmitted in an epidemic cycle between *Aedes* mosquitoes and humans, as well as through sexual and *in utero* transmission. Most of the studies reporting ZIKV sexual transmission indicate that male-to-female is the most common route of transmission [1,2,3,4,5], although female-to-male [6] and male-to-male [7] have also been reported. Even though sexual transmission of ZIKV can only be confirmed when infection takes place in *Aedes* free areas, epidemiological studies indicate that women are more likely to be infected by ZIKV during outbreaks [8,9] in the endemic regions, which could be via male-to-female sexual transmission. Additionally, supporting the role of sexual transmission is the fact that the infectious ZIKV can be found in semen up to 69 days post-infection [2,10,11,12,13,14,15,16], as well as in the vaginal secretion of infected woman [17,18]. These findings provide strong evidence for the role of sexual transmission in ZIKV dissemination.

Several studies have demonstrated that vaginal ZIKV replication and sexual transmission during early pregnancy can lead to birth defects in mice [19,20,21,22]. However, most of these studies used immune compromised animals, mainly because a limited ZIKV-related pathology was detected in the wild type animals. For instance, wild-type C57BL/6 mice intravaginally infected with ZIKV showed only transient viremia (7 days), followed by a complete recovery [19]. Although embryos from these dams developed growth defects, virus RNA was not detected in the placenta and in the fetuses [19]. While studies using immunodeficient animals were essential for the understanding of ZIKV infection mechanisms, these models do not recapitulate the pathology in humans. For instance, most of these animals succumb to infection a few days after the exposure [19,23]. This is in contrast to what is commonly found in humans, where the majority (about 80%) of the infected adult patients are asymptomatic, and most of the remaining individuals exhibit only mild symptoms, including fever, headache, cutaneous rash, conjunctivitis, fatigue and/or arthralgia [9]. Moreover, these immune compromised animal models of ZIKV infection present with maternal encephalitis, which was not shown as being required for ZIKV teratogeny in humans [24,25]. Therefore, studies employing immune competent mouse models could provide more reliable results regarding ZIKV teratogeny in humans.

It was demonstrated that when pregnant wild-type FVB/NJ mice are injected via the jugular vein, ZIKV infects the maternal tissues, placentae and embryos [26]. Interestingly, embryos from FVB/NJ dams that were subjected to ZIKV infection during early pregnancy displayed severe malformations and a delayed development, as well as dysraphia and hydrocephalus, resembling microcephaly in newborns [26]. However, it remains unknown whether the vaginal infection of these immunocompetent mice would cause viremia and fetus infection. Furthermore, our understanding of the cytokine activation pattern following intravaginal ZIKV infection in dams and fetuses remains limited, as most employed animal models are immunocompromised. Therefore, in this study, we sought to investigate whether the ZIKV intravaginal infection of FVB/NJ immunocompetent dams during early pregnancy would lead to a placenta and embryo brain infection. We also investigated the host immune response to ZIKV by measuring the expression of inflammatory factors.

## 2. Materials and Methods

### 2.1. Animals

FVB/NJ mice were purchased from the Jackson Laboratory (Bar Harbor, ME, USA). The mice were bred and housed in a controlled room at 23 °C on a 12 h light/12 h dark cycle. All animal procedures were approved by the Committee on Animal Ethics of the Universidade Federal de Minas Gerais (CEUA/UFMG, permit protocol 400/2018).

### 2.2. Virus

A low-passage-number clinical isolate of ZIKV (HS-2015-BA-01), obtained from a viremic patient with a symptomatic infection in Bahia State, Brazil, in 2015, was used. The complete genome of the virus is available at GenBank under the accession no. KX520666. Virus stocks were propagated in C6/36 *Aedes albopictus* cells and titrated as described previously [27].

### 2.3. Intravaginal Infection

Vaginal lavage was collected using 20 µL of sterile PBS and transferred to a glass slide to determine the cell morphology by light microscopy. Two female mice (2 to 4 months of age) in the estrus phase were placed with one male for a period of 24 h in order to generate timed pregnant females. Four days after intercourse, another vaginal lavage was done to establish the pregnancy. Females identified as pregnant were inoculated with 10 µL of ZIKV (1.0 × 10^5^ PFU) or PBS (MOCK infection) on the gestational day (GD) 4.5. Females’ weight was determined on GD 4.5, 11.5 and 17.5. The dams and embryo tissues were collected on GD 17.5.

### 2.4. Quantitative PCR (qPCR)

The total RNA was isolated from the tissues using Trizol™ reagent, according to the manufacturer’s instructions (Thermo Scientific, Waltham, MA, USA). An aliquot of total RNA (1 µg) was used for the cDNA synthesis (Superscript kit; ThermoFisher Scientific). cDNA (1 μL for each target) was used for the relative quantification of transcripts in a qPCR assay. The ∆Ct values were calculated by normalizing with the respective GAPDH Ct values, and the fold changes were calculated using the ∆∆Ct method relative to the mock-infected control cells. Each value represents the average of three experiment replicates. The primer sequences are summarized in Table 1.

### 2.5. Statistical Analysis

The means ± SEM are shown for the number of tested animals indicated in the figures. GraphPad Prism™ software was used to analyze the data for statistical significance. The statistical significance (*p* < 0.05) was determined either by a t-test (when only two groups were analyzed) or through an analysis of variance (ANOVA) testing followed by a Tukey’s multiple comparisons test.

## 3. Results

**Figure 1:**
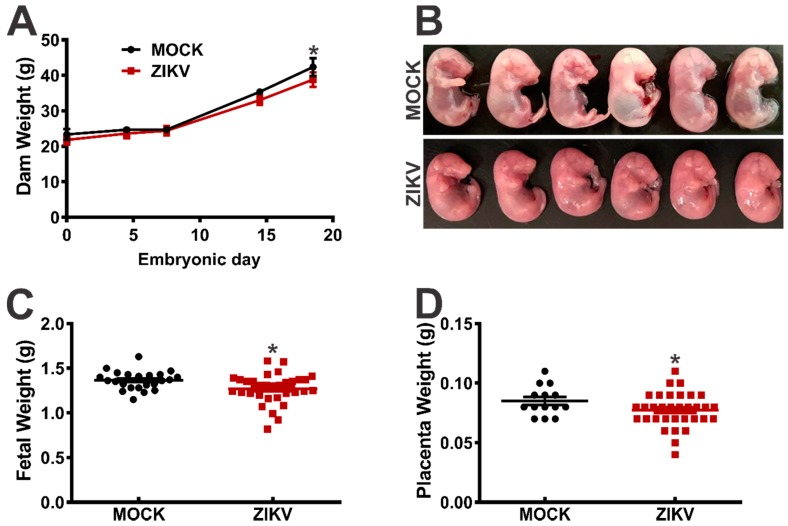
The ZIKV effect on FVB/NJ mice dams and fetuses. The FVB/NJ mice were infected intravaginally with 10 µl of ZIKV (1.0 × 10^5^ PFU) or PBS (MOCK infection) on the gestational day (GD) 4.5. The dam weight was determined on the GDs 4.5, 11.5 and 17.5. (**B**) Representative images of embryos derived from either the MOCK- or ZIKV-infected dams are shown. The graphs show the weight of the (**A**) dams, (**C**) fetuses and (**D**) placentae. The data represent the means ± SEM. * indicates a significant difference, as compared to MOCK (*p* < 0.05).

To develop an immunocompetent mouse model of ZIKV infection, we challenged pregnant FVB/NJ mice with either PBS (MOCK infection) or ZIKV (1.0 × 10^5^ PFU) intravaginally, on GD 4.5. The dams were weighed on GD 4.5, 11.5 and 17.5. Although the maternal weight did not differ at the early stages of pregnancy, the weight of the ZIKV-infected dams was significantly lower at GD 17.5, as compared to that of the MOCK-infected animals (Figure 1A). Moreover, the weight of the fetuses (Figure 1B,C) and placentae (Figure 1D) collected from the ZIKV-infected females was lower, as compared to the MOCK-infected controls. Furthermore, among the 26 fetuses derived from the ZIKV-infected dams, two were reabsorbed (7.7%), while all 31 embryos from the MOCK-infected females were intact with only one of the fetuses showing signs of malformation (3.2%) (Table 2, Figure 1B). Additionally, it appears that ZIKV infection did not affect the number of fetuses, as it was similar between the ZIKV-infected (8.6 ± 1.3) and MOCK-infected (7.0 ± 1.2) dams.

Table 2 shows the number of dams employed in this study, as well as the number of embryos obtained from the ZIKV-positive and MOCK pregnancies that were malformed or reabsorbed. 

The brain and spleen of dams 3 through 7 (Table 2), as well as the placenta and brain from their respective fetuses, were collected on GD 17.5 to determine the ZIKV transcripts and the levels of inflammatory factors. The ZIKV infection of dams was confirmed in the spleen, although the brains were not infected by the virus (data not shown).

For the gene expression analysis, 14 embryos from the ZIKV-infected dams were compared to 21 embryos from the MOCK-infected females (Table 2). Interestingly, most of the analysed placentae from the ZIKV-infected females were positive for ZIKV (13 out of 14, Table 4), although the brain of only 6 of these embryos were positive for ZIKV (Table 3). These data indicate that the placenta could serve as an efficient barrier preventing virus entrance, even in the case of an ascending intravaginal infection. Next, we decided to analyse the expression of inflammatory genes in the embryos from the ZIKV-infected dams that were either negative or positive for ZIKV in the brain (Table 3). These data could help in determining whether a virus presence in the developing neural tissue would be necessary to elicit a local inflammatory response or whether the infection of dams would be sufficient to induce neuroinflammation even when ZIKV could not reach fetal neural tissue.

Table 3 shows the number of embryos that exhibited an increased brain expression of inflammatory genes (TLR7, Mx1, CCL2, CXCL1, CXCL10, IL-1β, IL-18, TFN-α and IL-6) in ZIKV-positive (6 out of 14) or negative (8 out of 14) brains. All 14 tested embryos are derived from ZIKV-infected dams.

Table 4 shows the number of embryos that exhibited an increased placenta expression of inflammatory genes (TLR7, Mx1, CCL2, CXCL1, CXCL10, IL-1β, IL-18, TNF-α and IL-6) in ZIKV-positive (13 out of 14) or negative (1 out of 14) placentae. All 14 tested embryos are derived from ZIKV-infected dams.

In order to understand the host response to virus infection and the potential neuroinflammatory consequences of ZIKV infection, we analyzed the expression of genes involved in virus recognition and elimination, as well as in inflammation in the placenta and fetal brain. The data on the gene expression levels in the brain of the ZIKV-positive fetuses (Supplementary Figure S1) and ZIKV-negative fetuses (Supplementary Figure S2) collected from the ZIKV-infected dams are presented. To make the results easier to understand, the data shown on Supplementary Figures S1 and S2 were summarized in Table 3 (brain samples) and Table 4 (placenta samples).

**Figure 2:**
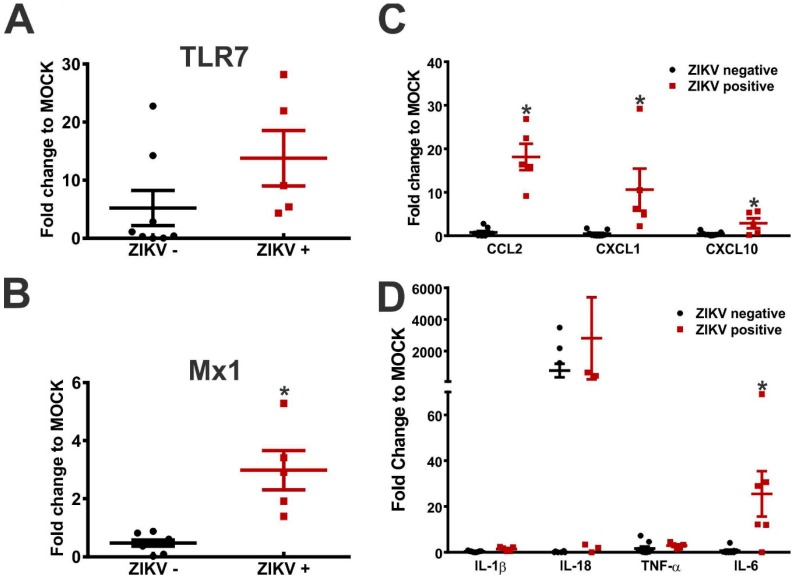
The ZIKV presence in the brain tissue modifies the expression of inflammatory factors in the brains collected from embryos derived from the ZIKV-infected FVB/NJ dams. Fetus brains were collected on GD 17.5 and used to extract total RNA. cDNA was amplified and used for a qPCR analysis. The real-time PCR values were normalized to those of GAPDH of the corresponding samples. The relative levels for each gene were calculated in reference to the mock-infected cells using the ∆∆Ct method. The graphs show (**A**) TLR7, (**B**) Mx1, (**C**) CCL2, CXCL1 and CXCL10, and (**D**) IL-1β, IL-18, TNFα and IL-6 gene transcription levels (fold change to MOCK) in brains that were either positive or negative for ZIKV and that were derived from the ZIKV-infected FVB/NJ dams. The data represent the means ± SEM obtained from 6 ZIKV-positive brains and 8 ZIKV-negative brains. * indicates a significant difference, as compared to the ZIKV-negative brains (*p* < 0.05).

The toll-like receptor 7 (TLR7), a specialized sensor that recognizes RNA viruses and triggers the antiviral response, has been implicated in the regulation of ZIKV infection [28,29]. Interestingly, the TLR7 expression was increased in most of the brains (10 out of 14, Table 3) and placentae (11 out of 14, Table 4) of the embryos collected from the ZIKV-infected dams, as compared to those of the MOCK-infected animals, regardless of the presence of ZIKV in the analyzed tissue. Interestingly, the TLR7 level increased even in the case of one embryo whose placenta and brain were both ZIKV-negative (Table 3 and Table 4 and Supplementary Figure S3E). We have also tested whether the presence of ZIKV in the brain is essential for the observed changes in TLR7 expression. For that, the TLR7 levels were compared between the ZIKV-positive and negative brains of embryos derived from the ZIKV-infected dams. Interestingly, the TLR7 levels were not different between the ZIKV-positive and -negative mouse brains (Figure 2A). These data suggest that TLR7 expression could be induced in ZIKV-negative tissue by factors others than virus antigens.

It was shown that the type 1 interferon (IFN) responses, which can be achieved through TLR7/9 activation, can attenuate the ZIKV infection and consequent neurological alterations [19,20,30,31]. Mx1 is a type 1 IFN response gene capable of inhibiting viral RNA transcription and replication [32]. Thus, we measured the Mx1 level in fetuses from the MOCK- and ZIKV-infected dams. Mx1 mRNA was elevated in the brain of most fetuses that were positive for ZIKV (5 out of 6, Table 3). Interestingly, 1 fetal brain that was ZIKV-negative also had an increased level of Mx1, as compared to MOCK (Table 3). Regarding the placenta, 5 out of 13 placentae that were positive for ZIKV showed an augmented level of Mx1 (Table 4). To evaluate whether the presence of ZIKV in the brain was important for an increased Mx1 expression, we compared the mRNA levels between the ZIKV-positive and negative brains. The Mx1 levels were significantly increased in the ZIKV-positive, as compared to the ZIKV-negative brains (Figure 2B), indicating that ZIKV presence in the brain tissue is essential for an increased Mx1 expression.

An increased expression of cytokines and chemokines is often observed following viral infection [33]. Therefore, we examined the effect of ZIKV on the cytokine and chemokines transcript levels. CCL2 levels were increased in all 6 ZIKV-positive embryo brains and also in 2 brains from the ZIKV-negative embryos, as compared to the MOCK levels (Table 3). However, only 7 of the 13 infected placentae revealed augmented levels of CCL2 (Table 4). Similar to CCL2, the CXCL1 levels were increased in the brain of all 6 ZIKV-positive embryos, as compared to MOCK (Table 3). However, the CXCL1 expression did not increase in any of the ZIKV-negative embryo brains (Table 3). Moreover, CXCL1 increased in 7 out of 13 infected placentae (Table 4). The CXCL10 levels were not affected in the ZIKV-negative brain samples, but were specifically elevated in the ZIKV-positive embryo brains (Table 3), as well as in infected placentae (Table 4), as compared to MOCK. Importantly, the levels of these three chemokines were significantly enhanced in the ZIKV-positive, as compared to the ZIKV-negative fetal brains (Figure 2C), indicating that the presence of ZIKV in the fetal brain is necessary to enhance the gene expression of these chemokines.

Regarding inflammatory cytokines, although several embryos from ZIKV-infected mothers showed augmented cytokine levels, in most cases this increase was not exclusively associated with ZIKV in the brain tissue (Table 3). The IL-1β levels increased in the brain of 3 embryos that were positive for ZIKV (Table 3) and also in 2 placentae that were infected with ZIKV (Table 4), as compared to MOCK. Although the IL-1β gene expression only increased in the ZIKV-positive tissue, the average levels were not different when comparing embryo brains that were positive for ZIKV to those that were negative for the virus (Figure 2D). In contrast, the IL-18 levels were increased in most brains (8 out of 14, Table 3) and placentae (10 out of 14, Table 4) of fetuses from the ZIKV-infected dams, regardless of the ZIKV presence in the tissue. For instance, the IL-18 levels were increased in the only ZIKV-negative placenta (Table 4). Moreover, the IL-18 expression was increased in 5 ZIKV-positive and in 3 ZIKV-negative brains, as compared to MOCK (Table 3). However, when the IL-18 mRNA levels were compared between the ZIKV-positive and negative brain samples, no difference was observed (Figure 2D). The TNF-α levels were upregulated in 6 ZIKV-infected placentae (Table 4) and in 5 ZIKV-positive and in 2 ZIKV-negative brains (Table 3), as compared to MOCK. Similar to the IL-18, the levels of TNF-α did not differ between the ZIKV-positive and negative embryo brains (Figure 2D). In contrast, the IL-6 levels were more elevated in the ZIKV-positive than in the ZIKV-negative brains (Figure 2D). The IL-6 levels increased in 8 ZIKV-positive placentae (Table 4), as well as in 5 ZIKV-positive brains and in one ZIKV-negative brain (Table 3), as compared to MOCK. Therefore, an increased expression of the tested inflammatory cytokines appears to rely more on the ZIKV infection of dams than on the presence of ZIKV in the tested brain tissue.

## 4. Discussion

ZIKV infection of pregnant women can lead to congenital Zika syndrome (CZS), which is characterized by severe microcephaly, a partially collapsed skull, decreased brain tissue with a specific pattern of brain damage and calcifications, eye alterations, congenital contractures and hypertonia restricting body movements [34]. However, only a small fraction (5 to 15%) of ZIKV-positive pregnant women give birth to babies exhibiting CZS [35,36,37]. Therefore, it is plausible that additional factors contribute to ZIKV teratogeny. For instance, poor living conditions have been suggested as an important factor contributing to ZIKV-related birth defects [38]. Supporting this assumption, about 90% of the microcephaly cases in Brazil were registered in the northeast of Brazil, a region that has faced many socio-economic problems and poor nutrition over decades [39]. However, the high prevalence of ZIKV infection in these areas, which are overcrowded and lack basic sanitation, could only partially explain the correlation between the high prevalence of CZS and poor living conditions. Thus far, it remains to be determined whether sexual transmission could contribute to a higher risk of congenital infection as compared to mosquito-borne transmission.

Interestingly, it has been shown that the percentage of ZIKV positive fetuses was higher when immunodeficient (AG129) female mice were infected sexually (88%), as compared to sub-cutaneous (50%) or intravaginal (53%) injection [40]. These data suggest that sexual transmission could account for some of the ZIKV-induced teratogenic cases observed within *Aedes* endemic regions. Based on these results and based on the data we presented here, we propose that sexual transmission could be another factor contributing to CZS. Corroborating this hypothesis, we demonstrated that about 42% of embryos collected from immunocompetent wild-type FVB/NJ females submitted to ZIKV intravaginal infection were positive for the virus in the brain. In addition, we observed increased levels of neuroinflammatory factors in the brain of fetuses derived from ZIKV-infected dams. Interestingly, the alterations in the levels of the chemokines CCL2, CXCL1 and CXCL10, as well as the changes regarding IL-6 and Mx1, were linked to ZIKV-positive fetuses, indicating that infected fetuses exhibit a robust inflammatory immune response that is dependent on the virus presence in the analyzed tissue and independent of the mother’s response to ZIKV. These results are similar to what was found in ZIKV-infected pregnant rhesus macaques, where infected fetuses exhibited a robust immunological response to ZIKV infection [41]. Therefore, by using immunocompetent wild-type FVB/NJ mice intravaginally challenged with ZIKV, we have established a mouse model to study ZIKV-related CZS that could more reliably recapitulate the neurological alterations observed in human newborns.

Corroborating previous publications, our findings indicate that ZIKV must cross gestational barriers before reaching the brain of the fetus. The placenta appears as a very important barrier, as we observed that most placentae (93%) were infected by ZIKV, although only about 42% of the embryos exhibited brain infection. These data indicate that even in an ascending vaginal infection, ZIKV may reach the conceptuses through the placenta and that placenta appears to interfere with the viral access to embryos and fetuses. Moreover, we observed that fetuses from infected dams displayed an anti-viral response, even when ZIKV is not present in the tested fetal tissue. For instance, the expression of TLR7 was increased in the brain of fetuses from infected dams, regardless of ZIKV presence in the fetal brain tissue. As TLR7 is mainly activated by single stranded RNA [42], we hypothesize that this increase in the TLR7 expression could lead to an enhanced activation of anti-viral cell signaling pathways, limiting ZIKV infection. Notably, it has been shown that an agonist of TLR7/8 blocks ZIKV replication in human monocytes by inducing the antiviral protein viperin [29]. It is documented that fetuses can be protected from vertical transmission of viruses through the induction of a robust antiviral response without excessive production of pro-inflammatory factors or the recruitment of inflammatory cells [43,44]. The activation of TLR7 can increase the levels of type 1 IFN, which consequently upregulates the expression of genes capable of inhibiting viral replication, degrading viral nucleic acids, and inducing an antiviral state [45].

It appears that the anti-viral response is protective against ZIKV, as mice lacking a type I IFN response are more susceptible to ZIKV infection and develop neurological alterations [19,20,31]. Furthermore, it has been shown that IFN-λ administration during mid-pregnancy suppresses ZIKV replication and protects the fetus by upregulating Mx1 [30]. We have shown in this study that Mx1 expression is increased in ZIKV positive fetuses. As Mx1 inhibits viral RNA transcription and replication [32], we suggest that the activation of the TLR7/IFN/Mx1 cell signaling pathway could help eliminate ZIKV from the brain of some of the fetuses whose placentae were ZIKV-positive and whose brains were ZIKV-free by the time of tissue collection (GD17.5).

We also observed increased levels of several inflammatory factors in the brain of fetuses collected from the ZIKV-infected dams, which could also have a protective effect contributing to viral clearance [33,46]. Corroborating this hypothesis, TLR7 and IL-18 expression levels were found elevated in some of the ZIKV-negative brains, possibly because these brains were positive for ZIKV at earlier gestational days. Another possibility is that the maternal response to ZIKV could be triggering this increase in inflammatory factors in the brain of ZIKV free fetuses. Future experiments will be important for verifying these hypotheses experimentally. Moreover, it is also important to mention that a strong type 1 IFN response is often accompanied by the recruitment of inflammatory cells, including neutrophils, inflammatory monocytes, natural killer (NK) and T lymphocytes, which can potentially lead to brain tissue injury [47]. Importantly, we have shown that ZIKV activates inflammasomes in monocytes, which could lead to local inflammation and tissue damage [48].

Increased transcriptional levels of the chemokines CCL2, CXCL1 and CXCL10 in the brain of ZIKV-positive embryos could indicate the increased recruitment of inflammatory cells, facilitating viral elimination, but also exacerbating neuroinflammation. Interestingly, it has been shown that CCL2 levels are increased in ZIKV-infected pregnant women [49]. Notably, CCL2 was found to be the most highly induced inflammatory factor in ZIKV-positive pregnant women with abnormal birth outcomes, as compared to that of ZIKV-positive pregnant women without birth complications [49]. Corroborating these results, it has been shown that CCL2 levels were found to be increased in the amniotic fluid of ZIKV-positive women giving birth to babies with neonatal microcephaly, relative to uninfected control group [50]. Similarly, CXCL10, a chemokine that has been associated with neuronal damage, was the most highly induced inflammatory factor in symptomatic ZIKV-positive pregnant women and was also specifically elevated in pregnant women with abnormal birth outcomes [49,51]. Therefore, the elevated levels of these chemokines could be playing a detrimental role in ZIKV-infected embryo brains.

## 5. Conclusions

The intravaginal ZIKV infection model presented here will be an excellent tool for studying ZIKV-linked neuroinflammatory alterations. The immunocompetent mouse strain, FVB/NJ, will be suitable for studying the role of inflammatory factors that would otherwise be impossible to study when using genetically modified immunodeficient animals. For instance, the increased levels of CCL2 and CXCL10 observed in human subjects could be recapitulated in wild type FVB/NJ mice challenged with ZIKV into the vagina. Moreover, this mouse model of ZIKV-induced CZS could help to determine whether an ascending infection from the vagina to the fetus may provide an easier path for ZIKV to reach the fetus, as compared to through a mosquito bite.

## Figures and Tables

**Table 1:** Primer sequences.

Primer Target	Forward	Reverse
IL-1β	AGCTTCAAATCTCGCAGCAG	TCTCCACAGCCACAATGAGT
Il-6	GACTGATGCTGGTGACAACC	AGACAGGTCTGTTGGGAGTG
IL-18	CTTCTGCAACCTCCAGCATC	GTGAAGTCGGCCAAAGTTGT
Ccl2	AACTGCATCTGCCCTAAGGT	CTGTCACACTGGTCACTCCT
Cxcl1	TGTGGGAGGCTGTGTTTGTA	ACGAGACCAGGAGAAACAGG
Cxcl10	AGCCATGGTCCTGAGACAAA	ACAGAGCTAGGACAGCCATC
Mx1	AGGCAGTGGTATTGTCACCA	AGACTTTGCCTCTCCACTCC
TLR7	ATGTCCTTGGCTCCCTTCTC	ACTGAGCCATGTCTCTTGCT
TNFα	CTCATGCACCACCATCAAGG	ACCTGACCACTCTCCCTTTG
zika virus 1087-1163	CCGCTGCCCAACACAAG	CCACTAACGTTCTTTTGCAGACAT

**Table 2:** ZIKV- and MOCK-infected dams and litters.

Dam	ZIKV	*n* Embryos	Malformed	Reabsorptions	Total Dead
Dam 1	–	7	0	0	0
Dam 2	+	10	0	1	1
Dam 3	–	10	0	0	0
Dam 4	–	7	1	0	1
Dam 5	–	4	0	0	0
Dam 6	+	10	0	0	0
Dam 7	+	6	0	1	1

**Table 3:** Increased expression of inflammatory factors by the brain of embryos from ZIKV-infected dams.

	ZIKV+ (*n* = 6)	ZIKV- (*n* = 8)	Total (*n* = 14)
TLR7	6/6	4/8	10/14
Mx1	5/6	1/8	6/14
CCL2	6/6	2/8	8/14
CXCL1	6/6	0/8	6/14
CXCL10	4/6	0/8	4/14
IL-1β	3/6	0/8	3/14
IL-18	5/6	3/8	8/14
TNF-α	5/6	2/8	7/14
IL-6	5/6	1/8	6/14

**Table 4:** Increased expression of inflammatory factors by the placenta associated with embryos from ZIKV-infected dams.

Dam	ZIKV+ (*n* = 13)	ZIKV- (*n* = 1)	Total (*n* = 14)
TLR7	10/13	1/1	11/14
Mx1	5/13	0/1	5/14
CCL2	7/13	0/1	7/14
CXCL1	7/13	0/1	7/14
CXCL10	3/13	0/1	3/14
IL-1β	2/13	0/1	2/14
IL-18	9/13	1/1	10/14
TNF-α	6/13	0/1	6/14
IL-6	8/13	0/1	8/14

## Supplementary Materials

Supplementary materials can be found at https://www.mdpi.com/1999-4915/11/6/558/s1.
