# Supplementary material for: Host Immune Response to ZIKV in an Immunocompetent Embryonic Mouse Model of Intravaginal Infection
Source: Viruses. 2019 Jun 17;11(6):558. doi: 10.3390/v11060558 (PMC6631669; doi:10.3390/v11060558)
Supplement: Supplementary file 1 [file viruses-11-00558-s001.pdf]

Supplementary material:

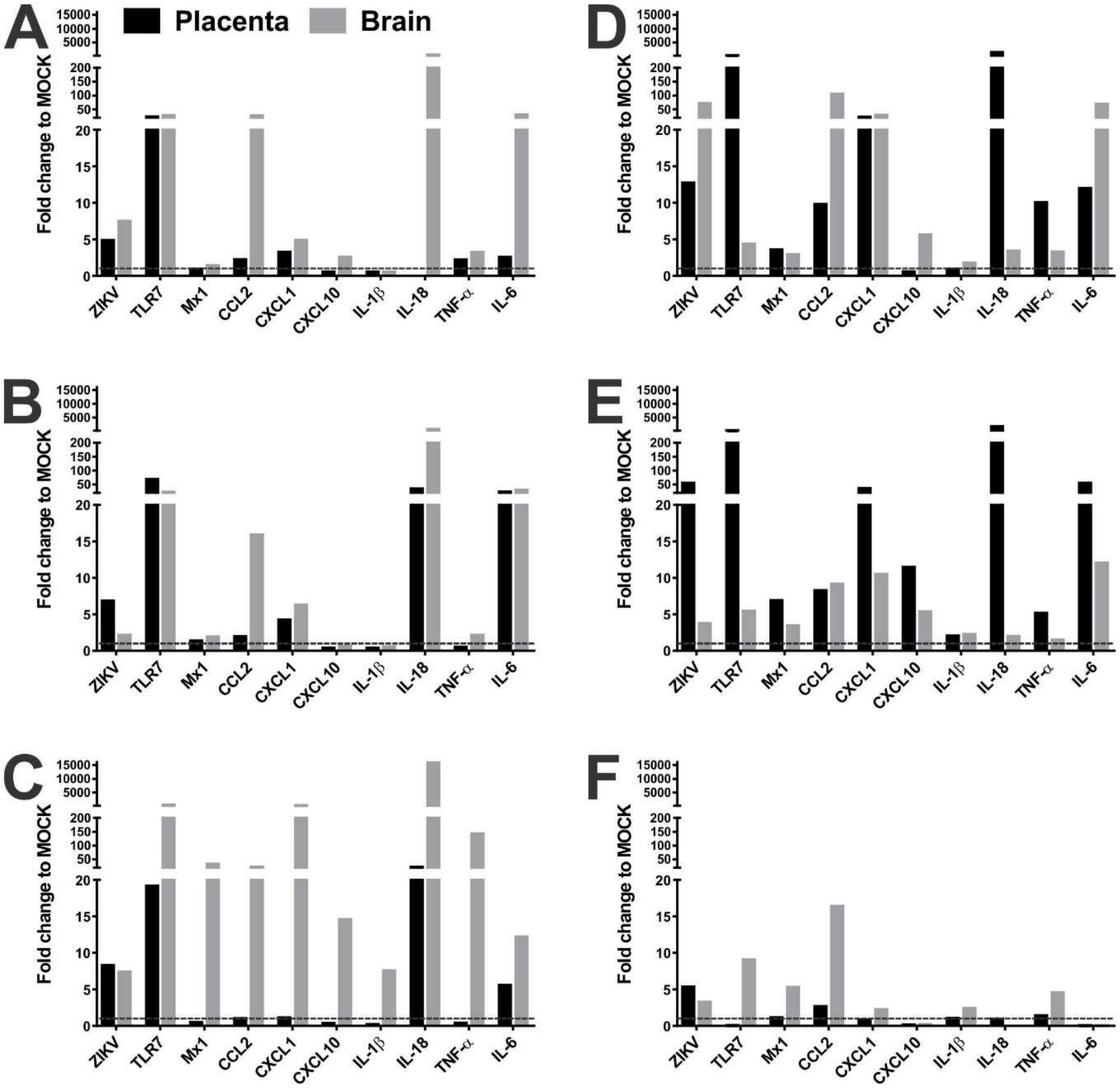

Supplementary figure 1. ZIKV effect on inflammatory factors gene transcription in placenta and brain of embryos positive for ZIKV in the brain. Placentae and fetus brains were collected on GD 17.5 and used to extract total RNA. cDNA was amplified and used for qPCR analysis. qPCR values were normalized to that of GAPDH of corresponding samples. Relative levels for each gene were calculated in reference to mock-infected embryos using ΔΔCt method. Graphs show TLR7, Mx1, CCL2, CXCL1, CXCL10, IL-1β, IL-18, TNFα, and IL-6 gene transcription levels and viral transcript accumulation (fold change to MOCK) in the placenta and brain of six embryos (A to F) that were derived from ZIKV-infected FVB/NJ dams and that were positive for ZIKV in the brain.

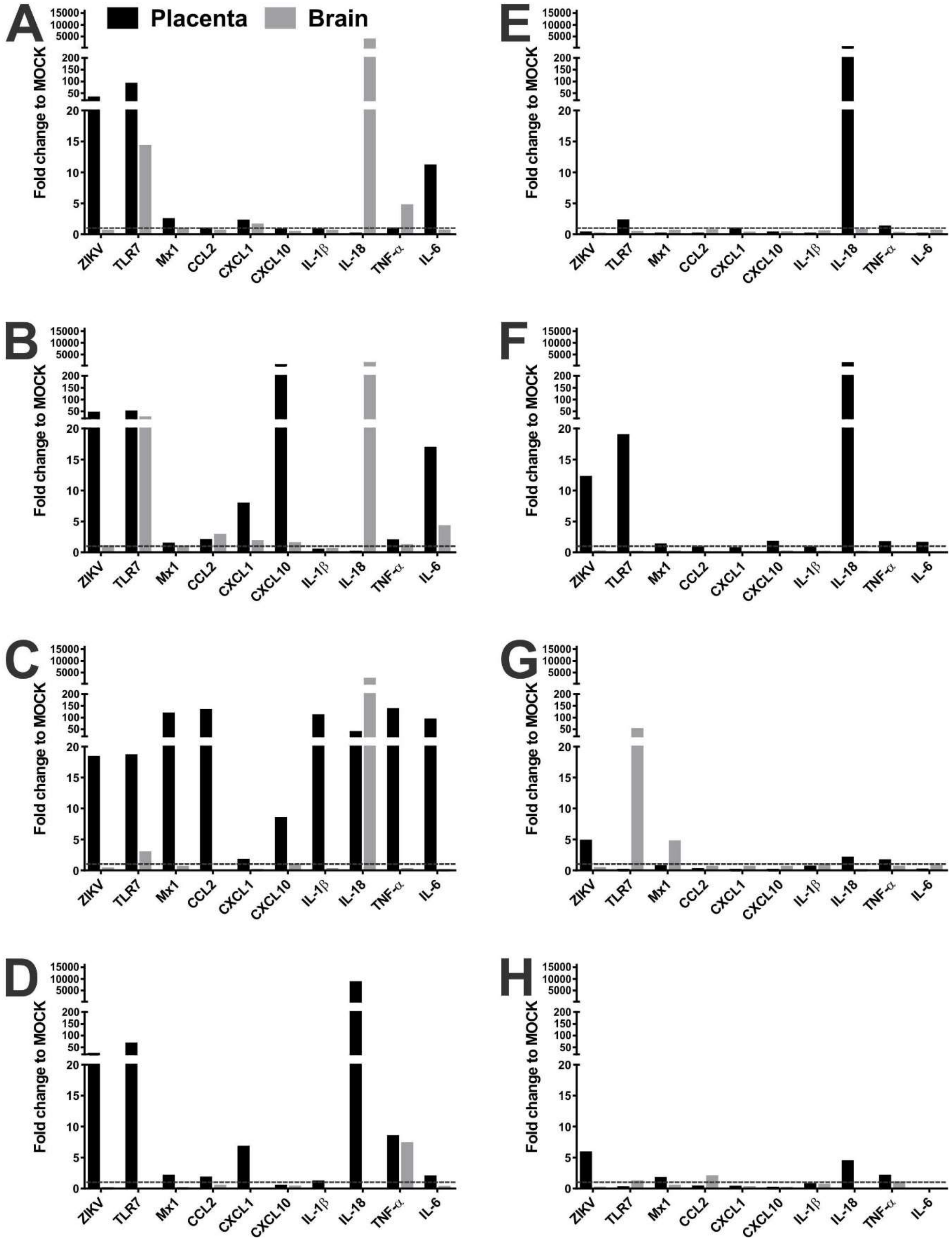

Supplementary figure 2. ZIKV effect on inflammatory factors gene transcription in placenta and brain of embryos negative for ZIKV in the brain. Placentae and fetus brains were collected on GD 17.5 and used to extract total RNA. cDNA was amplified and used for qPCR analysis. qPCR values were normalized to that of GAPDH of corresponding samples. Relative levels for each gene were calculated in reference to mock-infected embryos using ΔΔCt method. Graphs show TLR7, Mx1, CCL2, CXCL1, CXCL10, IL-1β, IL-18, TNFα, and IL-6 gene transcription levels and viral transcript accumulation (fold change to MOCK) in the placenta and brain of eight

embryos **(A-H)** that were derived from ZIKV-infected FVB/NJ dams and that were negative for ZIKV in the brain.
